# Risks and benefits of vaccines in diabetes

**DOI:** 10.1111/1753-0407.13481

**Published:** 2023-09-26

**Authors:** Zachary Bloomgarden

**Affiliations:** ^1^ Department of Medicine, Division of Endocrinology, Diabetes and Bone Disease Icahn School of Medicine at Mount Sinai New York New York USA

We are approaching 4 years from the onset of the COVID‐19 pandemic, and it is appropriate to review the topic of recommended vaccines for persons with diabetes. Infections are important causes of adverse outcome among persons with diabetes and are associated with morbidity and mortality substantially above rates among persons not having diabetes. Analysis of >500 000 UK Biobank participants showed that diabetes was associated with 1.5‐fold and 1.8‐fold greater COVID mortality in women and in men, respectively, but also with 2.2‐fold and 1.9‐fold greater influenza/pneumonia mortality in women and in men.^1^ The 2014 US Medical Expenditure Panel Surveys, representing 24 million persons with and 218 million without diabetes, showed that diabetes was associated with nearly a doubling in the likelihood of pneumonia and with a 2.6‐fold increase in hospitalization for pneumonia.^2^ Diabetes was associated with a nearly 4‐fold increase in likelihood of herpes zoster,^3^ with these infections in turn associated with increases in myocardial infarction (MI) and stroke.^4^ The response of “social media” to COVID, however, has been associated with considerable misinformation about many healthcare recommendations.^5^ Many of my patients now question the benefit of routinely recommended vaccination to prevent influenza, COVID‐19, pneumonia, zoster, and the many other infections for which effective preventative measures are available. We will review some of these considerations and how they apply to the treatment of diabetes.

A number of vaccines are considered “must‐haves” for people with diabetes: vaccines for influenza, COVID‐19, hepatitis B, pneumococcal pneumonia, tetanus, and at age 50 or greater herpes zoster.^6^ New vaccines being developed may be appropriate, for example, that for respiratory syncytial virus (RSV).^7^

For influenza vaccination, manufacturer product information in studies of <2000 persons receiving vaccine versus placebo, most not having diabetes or other medical conditions, suggests moderate efficacy of 68% in reducing infection.^8^ There is evidence of benefit in at‐risk persons such as those with diabetes. An epidemiologic study of vaccinated persons with diabetes showed a 46% lower mortality than among those not vaccinated and an 11% lower rate of hospitalization for pneumonia.^9^ In two studies from Taiwan, one of persons with chronic kidney disease (CKD) not known to have underlying (Cardiovascular disease (CVD) showed that those who had received one, two, three, and four influenza vaccines between 1997 and 2008 had likelihood of acute coronary syndrome reduced 38%, 61%, and 87% in comparison to those who had not received the vaccine,^10^ and another study of persons with CKD showed > 50% reduction in progression to requirement for dialysis among those who had had the vaccine.^11^

A number of COVID‐19 vaccine preparations are now available, with administration currently recommended for all persons with diabetes.^12^ Although some evidence suggests greater likelihood of thrombotic and coronary events among persons having COVID with the Janssen and Oxford‐AstraZeneca vaccines, the mRNA vaccines were associated with reduction in such events,^13^ and a study from Hong Kong comparing 3218 unvaccinated persons with 5248 who had been vaccinated showed reduction in post‐COVID MI or stroke among persons with underlying CVD, with what appeared to be progressively greater reduction in such events with increasing number of doses either of an mRNA vaccine or a whole inactivated virus vaccine.^14^ There are population studies suggesting potential adverse events. In a study of events during the first 28 days after COVID‐19 mRNA vaccine among >6 million persons >65 years old between December 2020 and July 2021, deep vein thrombosis or pulmonary embolism occurred in 0.5%, MI in 0.1%, stroke in 0.05%, thrombocytopenic purpura in 0.05%, facial nerve palsy in 0.04%, and myocarditis or pericarditis in 0.01%; encephalomyelitis, transverse myelitis, or Guillain‐Barre syndrome occurred at a frequency of <1 per 100 000 vaccine recipients.^15^ The likelihood of such events in a comparator population not receiving the vaccine is uncertain, particularly for the very rare events, and for the more common CVD events the argument has been made that post‐COVID myocarditis and pericarditis is 1.39‐fold more common in unvaccinated than vaccinated persons, that MI was 26% less common in a UK study comparing vaccinated with unvaccinated persons age ≥ 70, and that MI and stroke risk were 52% and 60% lower, respectively, in a Korean study comparing vaccinated with unvaccinated persons.^16^

Other vaccines may be appropriate for persons with diabetes. The RSVpreF vaccine against RSV has been advocated based on the incidence of the infection of 3%–7%/year among older persons, leading in the United States to >150 000 hospitalizations annually, with a randomized controlled trial of >34 000 adults ≥age 60 showing 81% and 94% efficacy in reducing infection at ages 60–69 and 70–79, respectively, although with few infections requiring hospitalization.^7^ Although the initial report noted that three persons had had either Guillain–Barré syndrome or acute encephalitis,^7^ a recent update indicated that six cases of inflammatory neurologic events had occurred, leading the authors to caution, “RSV vaccination in older adults should be targeted to those who are at highest risk for severe RSV disease and therefore most likely to benefit.”^17^

The evidence for vaccines, then, is typically based on randomized controlled trials, which are usually underpowered to determine severe and uncommon adverse effects and even to determine true benefit in reducing hospitalization and mortality. Extrapolation from population studies may be flawed if the population receiving vaccine differs in unmeasured ways from the nonvaccinated population. Unless well‐designed trials are carried out of specific populations, in particular, of persons with diabetes, we may well continue to ask which vaccines truly are beneficial.

## References

[1] de Jong M , Woodward M , Peters SAE . Diabetes and COVID‐19–related mortality in women and men in the UK biobank: comparisons with influenza/pneumonia and coronary heart disease. Diabetes Care. 2021;44:e22‐e24. 33293345 10.2337/dc20-2378 PMC7818325

[2] Liu K , Lee GC . Healthcare utilisation and cost expenditures for pneumonia in individuals with diabetes mellitus in the USA. Epidemiol Infect. 2019;147:e212. doi:10.1017/S0950268819000979 31364575 PMC6624864

[3] Poirrier JE , Meyers JL , Nagar SP , Patterson BJ , Glasser LI , Jabbour SA . Herpes zoster incidence and burden in adults with type 2 diabetes in the U.S.: a retrospective database analysis. Diabetes Care. 2022;45:2585‐2593. 36149780 10.2337/dc21-2053 PMC9862293

[4] Minassian C , Thomas SL , Smeeth L , Douglas I , Brauer R , Langan SM . Acute cardiovascular events after herpes zoster: a self‐controlled case series analysis in vaccinated and unvaccinated older residents of the United States. PLOS Med. 2015;12(12):e1001919. doi:10.1371/journal.pmed.1001919 26671338 PMC4682931

[5] Abbasi K . Where now in the danse macabre of covid‐19 and misinformation? BMJ. 2023;382:p1884. doi:10.1136/bmj.p1884

[6] Handelsman Y , Anderson JE , Bakris GL , et al. DCRM multispecialty practice recommendations for the management of diabetes, cardiorenal, and metabolic diseases. J Diabetes Complicat. 2022;36(2):108101. 10.1016/j.jdiacomp.2021.108101 PMC9803322 34922811

[7] Walsh EE , Pérez Marc G , Zareba AM , et al. Efficacy and safety of a bivalent RSV prefusion F vaccine in older adults. N Engl J Med. 2023;388(16):1465‐1477. doi:10.1056/NEJMoa2213836 37018468

[8] Fluzone Quadrivalent product information. https://www.vaccineshoppe.com/assets/pdf/vsh/pi/fluzonehighdosequadrivalentpi.pdf

[9] Bechini A , Ninci A , Del Riccio M , et al. Impact of influenza vaccination on all‐cause mortality and hospitalization for pneumonia in adults and the elderly with diabetes: a meta‐analysis of observational studies. Vaccines. 2020;8:263. doi:10.3390/vaccines8020263 32486233 PMC7349976

[10] Chen CI , Kao PF , Wu MY , et al. Influenza vaccination is associated with lower risk of acute coronary syndrome in elderly patients with chronic kidney disease. Medicine. 2016;95:e2588. doi:10.1097/MD.0000000000002588 26844466 PMC4748883

[11] Sung L‐C , Chen C‐C , Liu S‐H , et al. Effect of influenza vaccination on the reduction of the incidence of chronic kidney disease and dialysis in patients with type 2 diabetes mellitus. J Clin Med. 2022;11(15):4520. 35956134 10.3390/jcm11154520 PMC9369464

[12] Nassar M , Misra A , Bloomgarden Z . (2023). COVID‐19 vaccination in persons with diabetes: How they work. In: Myers, AK , ed. Diabetes and COVID‐19. Contemporary Endocrinology. Springer, Cham. 10.1007/978-3-031-28536-3_13

[13] Botton J , Jabagi MJ , Bertrand M , Baricault B , Drouin J , Le Vu S , Weill A , Farrington P , Zureik M , Dray‐Spira R. Risk for myocardial infarction, stroke, and pulmonary embolism following COVID‐19 vaccines in adults younger than 75 years in France. Ann Intern Med 2022 175(9):1250‐1257. 35994748 10.7326/M22-0988 PMC9425709

[14] Ye X , Yan VKC , Yiu HHE , et al. BNT162b2 or CoronaVac vaccinations are associated with a lower risk of myocardial infarction and stroke after SARS‐CoV‐2 infection among patients with cardiovascular disease. J Am Heart Assoc. 2023;12:e029291. doi:10.1161/JAHA.122.029291 37119083 PMC10227224

[15] Harris DA , Hayes KN , Zullo AR , et al. Comparative risks of potential adverse events following COVID‐19 mRNA vaccination among older US adults. JAMA Network Open. 2023;6(8):e2326852. doi:10.1001/jamanetworkopen.2023.26852 37531110 PMC10398407

[16] Akhtar Z , Trent M , Moa A , Tan TC , Fröbert O , MacIntyre CR . The impact of COVID‐19 and COVID vaccination on cardiovascular outcomes. European Heart Journal. 2023;25(Suppl A):A42‐A49. doi:10.1093/eurheartjsupp/suac123 PMC10021497 36937372

[17] Melgar M , Britton A , Roper LE , et al. Use of respiratory syncytial virus vaccines in older adults: recommendations of the advisory committee on immunization practices–United States, 2023. MMWR. 2023;72:793‐801. doi:10.15585/mmwr.mm7229a4 37471262 PMC10360650

